# Impacts of vitrification on the transcriptome of human ovarian tissue in patients with gynecological cancer

**DOI:** 10.3389/fgene.2023.1114650

**Published:** 2023-03-17

**Authors:** Ruihuan Gu, Naidong Ge, Bin Huang, Jing Fu, Ying Zhang, Ningyi Wang, Yan Xu, Lu Li, Xiandong Peng, Yaoyu Zou, Yijuan Sun, Xiaoxi Sun

**Affiliations:** ^1^ Department of Shanghai Ji’ai Genetics & IVF Institute, Obstetrics and Gynecology Hospital, Fudan University, Shanghai, China; ^2^ Department of Female Fertility Preservation, Obstetrics and Gynecology Hospital of Fudan University, Shanghai, China; ^3^ Shanghai Key Laboratory of Female Reproductive Endocrine-Related Diseases, Obstetrics and Gynecology Hospital, Fudan University, Shanghai, China

**Keywords:** human, ovary, RNA sequencing, vitrification, transcriptome

## Abstract

**Objective:** This study investigated the effects of a vitrification/warming procedure on the mRNA transcriptome of human ovarian tissues.

**Design:** Human ovarian tissues were collected and processed through vitrification (T-group) and then subjected to RNA sequencing (RNA-seq) analysis, HE, TdT-mediated dUTP nick-end labeling (TUNEL), and real-time quantitative PCR, and the results were compared to those of the fresh group (CK).

**Results:** A total of 12 patients, aged 15–36 years old, with a mean anti-Müllerian hormone level of 4.57 ± 3.31 ng/mL were enrolled in this study. According to the HE and TUNEL results, vitrification effectively preserved human ovarian tissue. A total of 452 significantly dysregulated genes (|log2FoldChange| > 1 and *p* < 0.05) were identified between the CK and T groups. Among these, 329 were upregulated and 123 were downregulated. A total of 372 genes were highly enriched for 43 pathways (*p* < 0.05), which were mainly related to systemic lupus erythematous, cytokine–cytokine receptor interaction, the TNF signaling pathway, and the MAPK signaling pathway. *IL10, AQP7, CCL2, FSTL3,* and *IRF7* were significantly upregulated (*p* < 0.01), while *IL1RN, FCGBP, VEGFA, ACTA2,* and *ASPN* were significantly downregulated in the T-group (*p* < 0.05) compared to the CK group, which agreed with the results of the RNA-seq analysis.

**Conclusion:** These results showed (for the first time to the authors’ knowledge) that vitrification can induce changes in mRNA expression in human ovarian tissues. Further molecular studies on human ovarian tissues are required to determine whether altered gene expression could result in any downstream consequences.

## 1 Introduction

In China, 4.82 million new patients are annually diagnosed with cancer, which ranks first globally (Chen et al., 2016; Xia et al., 2022). According to the American Cancer Society, 927,910 adult females and 10,500 children were diagnosed with cancer in 2021 (Siegel et al., 2021). Advancements in cancer treatment have decreased overall cancer mortality. Specifically, the 5-year survival rates of women aged 15–39 and children aged ⩽14 years were 86.7% and 84%, respectively (Miller et al., 2020). Unfortunately, radiotherapy, chemotherapy, and age-related fertility decline can lead to premature ovarian insufficiency (POI) (Kasaven et al., 2022; Spath and Braat, 2019). Hence, the quality of life and reproduction protection needs to be urgently addressed. Fertility preservation (FP) is becoming an increasingly important field, and its demand is rapidly increasing.

FP methods include ovarian transposition, oocyte or embryo cryopreservation, and ovarian tissue cryopreservation (OTC) (Anbari et al., 2022). OTC is the only pre-treatment option currently available for FP in prepubescent and adult females who cannot undergo ovarian stimulation or a delay in oncological therapy (Handling et al., 2021; Herraiz and Cervello, 2020; Jing et al., 2021; Margita et al., 2022; International Society for Fertility Preservation–ESHRE–ASRM Expert Working Group, 2017; Practice Committee of American Society for Reproductive Medicine, 2014). Meanwhile, OTC can restore the reproduction function. Since the birth of the first baby born after thawing and transplantation of ovarian tissue (Donnez et al., 2004), OTC for FP has increased throughout the world. In December 2019, the American Society of Reproductive Medicine published a committee opinion that OTC should be an established medical procedure and should no longer be considered experimental (Koray et al., 2021; Practice Committee of American Society for Reproductive Medicine, 2019). A birth rate of approximately 35% can be achieved after auto-transplantation of cryopreserved ovarian tissue (Van der Ven et al., 2016), and pregnancies and live births have continued to exponentially increase (Khattak et al., 2022) reaching over 200 in 2020 (Dolmans et al., 2020). Ruan et al. (2022), from the Beijing Obstetrics and Gynecology Hospital, Capital Medical University, reported a patient with myelodysplastic syndrome who gave birth to a healthy girl after OTC, which was the first baby born in this manner in China.

Based on live birth outcomes after OTC and transplantation in humans, slow freezing is still considered the gold standard technique for OTC. Because of ice crystal formation (Donnez et al., 2013), many studies have evaluated the negative effects on ovarian tissues of the slow-freezing method, including higher levels of DNA fragmentation (Xiao et al., 2013), gene under-expression (Labrune et al., 2020), and metabolic and secretory issues (Einenkel et al., 2022). Recently, Kim et al. (2021) suggested that human OTC using a slow-freezing technique is associated with significant telomere shortening and altered senescence markers and mitochondrial structures (Boram et al., 2021). Vitrification offers advantages of time efficiency with no expensive equipment required, and more importantly, no formation of harmful ice crystals. Currently, vitrification has superseded the slow-freezing method for the cryopreservation of oocytes and embryos (Rienzi et al., 2017; Levi-Setti et al., 2016; Edgar and Gook, 2012). Overall, four successful deliveries have been reported from the vitrification and transplantation of human ovarian cortex (SilberDeRosa et al., 2018; Kawamura et al., 2013; Suzuki et al., 2015). However, there have been contradictory reports on vitrification (Fisch and Abir, 2018). Several studies on human ovarian tissue have shown that vitrification/warming has no effect on the induction of either the apoptotic process or the follicular pool, and it also increases the preservation of the ovarian stroma (Chang et al., 2011; Fabbri et al., 2014; Shi et al., 2017). However, some studies have indicated that slow freezing is superior to vitrification in terms of follicle survival and growth after transplantation (Lee et al., 2019; Terren et al., 2019).

Only a limited number of studies have evaluated the effectiveness of vitrification for OTC and transplantation, and more studies are needed to confirm the safety and relevance of the vitrification method before its use in clinical practice. RNA sequencing (RNA-seq), which measures global transcriptomics, has been used in oocytes after vitrification or *in vitro* maturation and provides informative transcriptomic changes in oocytes after different operations (Zhang et al., 2020; Gao et al., 2017; Jia et al., 2019). Therefore, the objective of this study was to analyze the effects of vitrification/warming on the global transcriptome of human ovarian cortex tissues using RNA-seq.

## 2 Materials and methods

### 2.1 Ethics

All patients provided written informed consent before vitrification. This study was approved by the Ethics Review Board of the Shanghai Ji Ai Genetics & IVF Institute of the Obstetrics and Gynecology Hospital of Fudan University in Shanghai (JIAI E2017-07).

### 2.2 Ovarian tissue collection and processing

Twelve patients who underwent laparoscopic surgery at the Department of Obstetrics and Gynecology Hospital of Fudan University from January 2018 to June 2021 were enrolled in this study. Among the 12 patients without a history of chemotherapy or radiotherapy, through pathological diagnosis, it was determined that nine patients had endometrial cancer, two had cervical cancer, and one had vulvar cancer.

A basic examination of the patients was performed. The pathological biopsy results showed no tumor ovarian metastasis, and the blood hormone test (including anti-Müllerian hormone (AMH), basal follicle-stimulating hormone, basal luteinizing hormone, and basal estradiol) and B-ultrasound examination showed normal ovarian function. Therefore, these patients were included in the study. Ovarian tissues were collected from the patients and were immediately immersed into GMOPS-PLUS (Vitrolife, Sweden AB), preheated to 4 °C and quickly transported to the laboratory on ice within 2 h. The tissues were cleaned, and the medulla was removed by dissection with small scissors. The cortex was cut into 15–20 (5 × 5 × 1 mm) strips for subsequent FP. For each patient, two tissue strips (3 × 3 × 1 mm) were randomly divided into fresh (CK) and vitrification/warming (T) groups.

### 2.3 Vitrification and warming procedure

For the vitrification of ovarian tissues, the Ova Cryo Kit Type M (Kitazato, Japan) was used as described by Suzuki et al. (2015). In brief, the Ova Cryo Kit Type M (Cryo1, Cryo2, and Cryo3) was equilibrated for 30 min at room temperature and vitrification was also performed at room temperature. The ovarian strips were first equilibrated in Cryo1 for 5 min, followed by equilibration in Cryo2 for 5 min. Subsequently, the strips were transferred to Cryo3 containing polyvinylpyrrolidone (PVP) for 15 min. Within 20 min, the ovarian strips were loaded with Ova Cryo Device Type M (ODT, open system) with a minimum volume of media and directly immersed into liquid nitrogen. For human ovarian tissue warming and after opening ODT in liquid nitrogen with tweezers and removing the vial, the strips were plunged into Thaw 1 and warmed to 37 °C in a water bath for 1 min. To remove the cryoprotectant, the ovarian strips were transferred from Thaw 1 into Thaw 2 at room temperature for 3 min, followed by incubation in Thaw 3 for 5 min. Afterward, the tissue was cultured in pre-warmed culture medium [McCoy’s 5a medium with glutamine supplemented with HEPES (Invitrogen Ltd., Paisley, United Kingdom), HAS (3 mg/mL), penicillin G (75 𝛍g/mL), and streptomycin (50 𝛍g/mL) (Sigma Chemicals, Poole, Dorset, United Kingdom)] for 4 h of recovery before the following analyses were conducted.

### 2.4 Morphological analysis of ovarian tissues before and after vitrification

The ovarian fragments in the CK and T groups were fixed in 4% paraformaldehyde (Sinopharm, China) for 4 h, dehydrated through a serial alcohol (Sinopharm, China) gradient, clarified with xylene (Sinopharm, China), embedded in paraffin wax, and serially sectioned into 5 𝛍m thickness. The ovarian sections were dewaxed in xylene, rehydrated through decreasing concentrations of ethanol, and stained with hematoxylin and eosin (Sigma, United States). After staining, the sections were dehydrated through increasing concentrations of ethanol and xylene. For the morphological evaluation, slides were examined using a microscope (Nikon, Japan) under ×200 magnification.

### 2.5 Apoptosis detection of ovarian tissues before and after vitrification

TdT-mediated dUTP nick-end labeling (TUNEL) was used to detect the follicle and interstitial cell apoptosis conditions using a FragEL™ DNA Fragmentation Detection kit (Merck, United States), according to the manufacturer’s protocol. After warming, the ovarian tissue sections were incubated with a labeling solution containing dUTP and an enzyme solution for 1.5 h at 37 °C and then counterstained with hematoxylin. The tissue sections were observed by fluorescence microscopy. When the cell nucleus was deeply stained with a brownish yellow, positive staining was observed.

### 2.6 RNA-seq analysis

**FIGURE 1:**
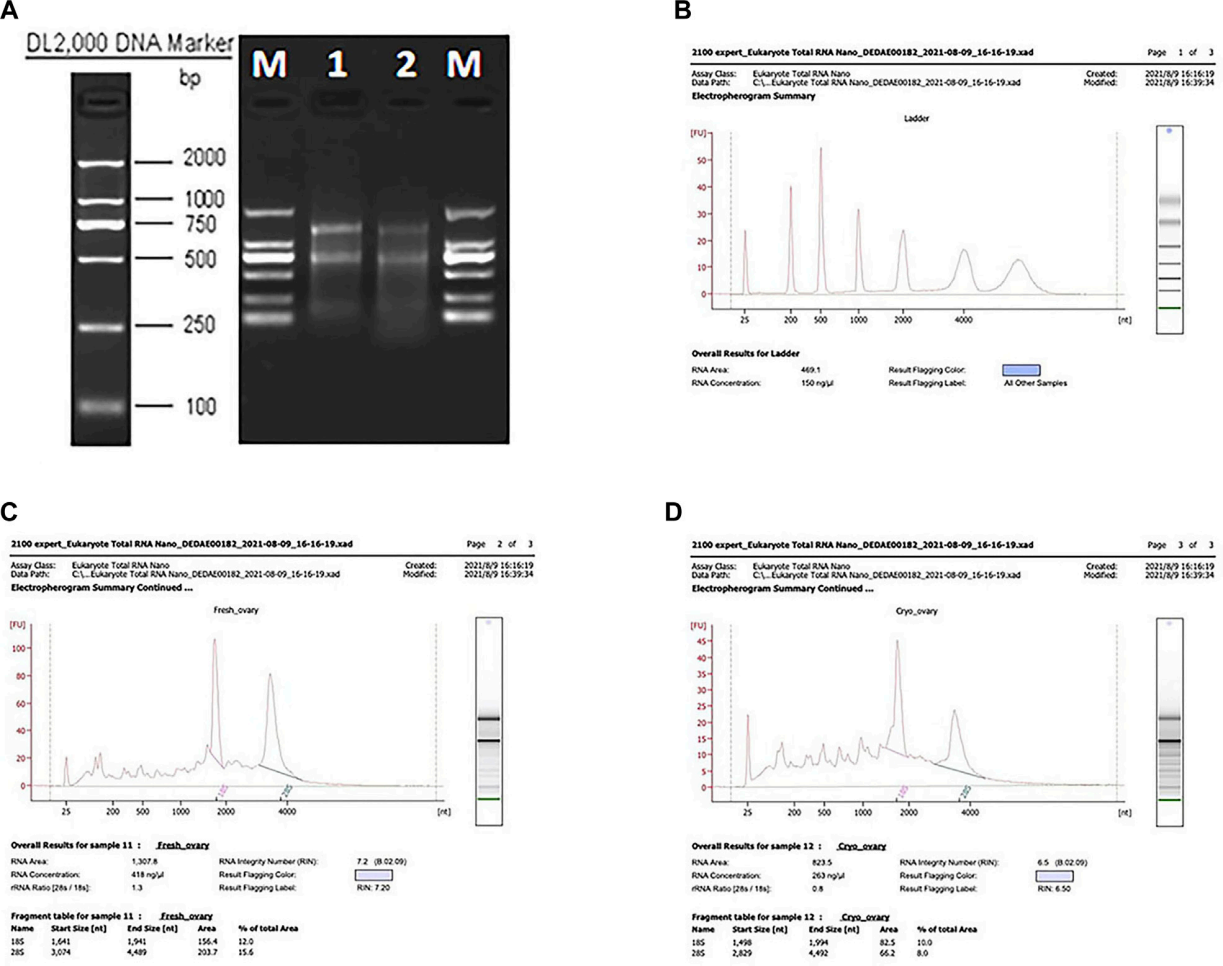
Results of the NanoDrop spectrophotometer, agarose gel electrophoresis, and the test using the Agilent 2100 instrument. **(A)** Agarose gel electrophoresis results. **(B–D)** RIN values of the CK and T groups.

Total RNA was isolated from the CK and T groups using the TRIzol reagent (Invitrogen, Carlsbad, CA, United States), following the manufacturer’s protocol. The concentration, quality, and integrity of total RNA were determined using a NanoDrop spectrophotometer (Thermo Fisher Scientific, United States), agarose gel electrophoresis (Biowest Agarose, Biowest), and an Agilent 2100 instrument (Agilent, United States) (Figure 1). According to the agarose gel electrophoresis results and RIN values, the RNA integrity and quality of the samples used in this study were satisfactory for the RNA-seq analysis. Overall, 3 𝛍g of RNA was used as the input material for the RNA sample preparations. Sequencing libraries were created using a TruSeq RNA Sample Preparation Kit (Illumina, San Diego, CA, United States). Briefly, mRNA was purified from the total RNA using poly T oligo-attached magnetic beads. Fragmentation was accomplished using divalent cations at elevated temperatures in an Illumina proprietary fragmentation buffer. First-strand cDNA was synthesized using random oligonucleotides and SuperScript II, and second-strand cDNA synthesis was subsequently performed using DNA polymerase I and RNase H. The remaining overhangs were converted into blunt ends through exonuclease/polymerase activities and the enzymes were removed. After adenylation of the 3′ ends of the DNA fragments, Illumina PE adapter oligonucleotides were ligated in preparation for hybridization. To select cDNA fragments of the preferred 200 bp in length, the library fragments were purified using the AMPure XP system (Beckman Coulter, Beverly, CA, United States). DNA fragments with ligated adapter molecules at both ends were selectively enriched using the Illumina PCR Primer Cocktail in a 15-cycle PCR reaction. The products were purified (AMPure XP system, United States) and quantified using an Agilent high-sensitivity DNA assay on a Bioanalyzer 2100 system (Agilent, United States). The sequencing library was sequenced on a HiSeq platform (Illumina, San Diego, CA, United States) by the Shanghai Personal Biotechnology Co. Ltd.

Samples were sequenced on the platform to obtain image files. We used Cutadapt (v1.15) software to filter the sequencing data and to obtain high-quality sequences (Clean Data) for further analysis, and the filtered reads were mapped to the reference genome using HISAT2 v2.0.5.

The read count values for each gene were compared using HTSeq (0.9.1) statistics as the original expression of the gene, which were standardized using fragments per kilobase per million (FPKM). Then, a difference expression of genes was analyzed by DESeq (1.30.0) with screened conditions as follows: an expression difference multiple∣log2FoldChange∣>1 with a significant *p*-value < 0.05. These FPKM values were used to draw the heatmap with the R language pheatmap (1.0.8). Gene Ontology (GO) and Kyoto Encyclopedia of Genes and Genomes Pathway (KEGG) enrichment analyses of differential genes were performed using topGO or clusterProfiler (3.4.4) software applications, respectively (the standard of significant enrichment is a *p*-value < 0.05).

### 2.7 Real-time quantitative PCR (RT-PCR)

Total RNA isolation was performed using the TRIzol reagent for each group. The RNA was quantified using a NanoDrop 2000 spectrophotometer (Thermo Fisher Scientific, Carlsbad, CA, United States). RNA samples were reversely transcribed using the PrimeScript™ RT Reagent Kit (Perfect Real-Time; TaKaRa BIO INC. Tokyo, Japan). Relative quantification was performed in triplicate using real-time PCR (ABI 7500 Real-Time PCR System, Applied Biosystems). Reactions were prepared using a mixture of Power SYBR Green PCR Master Mix (TaKaRa BIO INC., Tokyo, Japan) and 0.4–0.8 μM of each primer. For validation of RNA-seq results, 10 dysregulated genes, including five upregulated and five downregulated genes, were randomly chosen (*IL10, AQP7, CCL2, FSTL3, IRF7, IL1RN, FCGBP, VEGFA, ACTA2*, and *ASPN*). *GAPDH* (NM-001256799) was selected as the reference gene. Triplicate expression values of the other genes were set relative to *GAPDH via* the ΔΔCt method (Schmittgen and Livak, 2008; Kaether et al., 2006). The experiments were repeated at least three times. As a negative control, cDNA from no-template RT-PCR reactions was used. All qPCR data were analyzed using Prism 9 (version 9.0; GraphPad Software Inc. CA, United States). A statistical analysis was conducted using Student’s unpaired *t*-test, and data are represented as mean ± S.E.M.

### 2.8 Statistical analysis

SPSS 22.0 statistical software was used for the analysis. Qualitative data are expressed as percentages. Comparisons among the CK and T groups were performed using the *χ* ^2^-test; *p* < 0.05 was considered statistically significant.

## 3 Results

A total of 12 patients, aged 15–36 years old, with a mean AMH of 4.57 ± 3.31 ng/mL were enrolled in this study. For each patient, two tissue strips were randomly divided into the CK and T groups. The ovaries unfixed from six patients were used for RNA-seq, while the rest were used for RT-PCR. Meanwhile, 24 ovarian cortex tissue pieces with a size of 1 × 2 × 1 mm were cut from each tissue strip in the CK and T groups and fixed for HE and TUNEL analyses.

### 3.1 Morphological analysis of the human ovarian tissue

**FIGURE 2:**
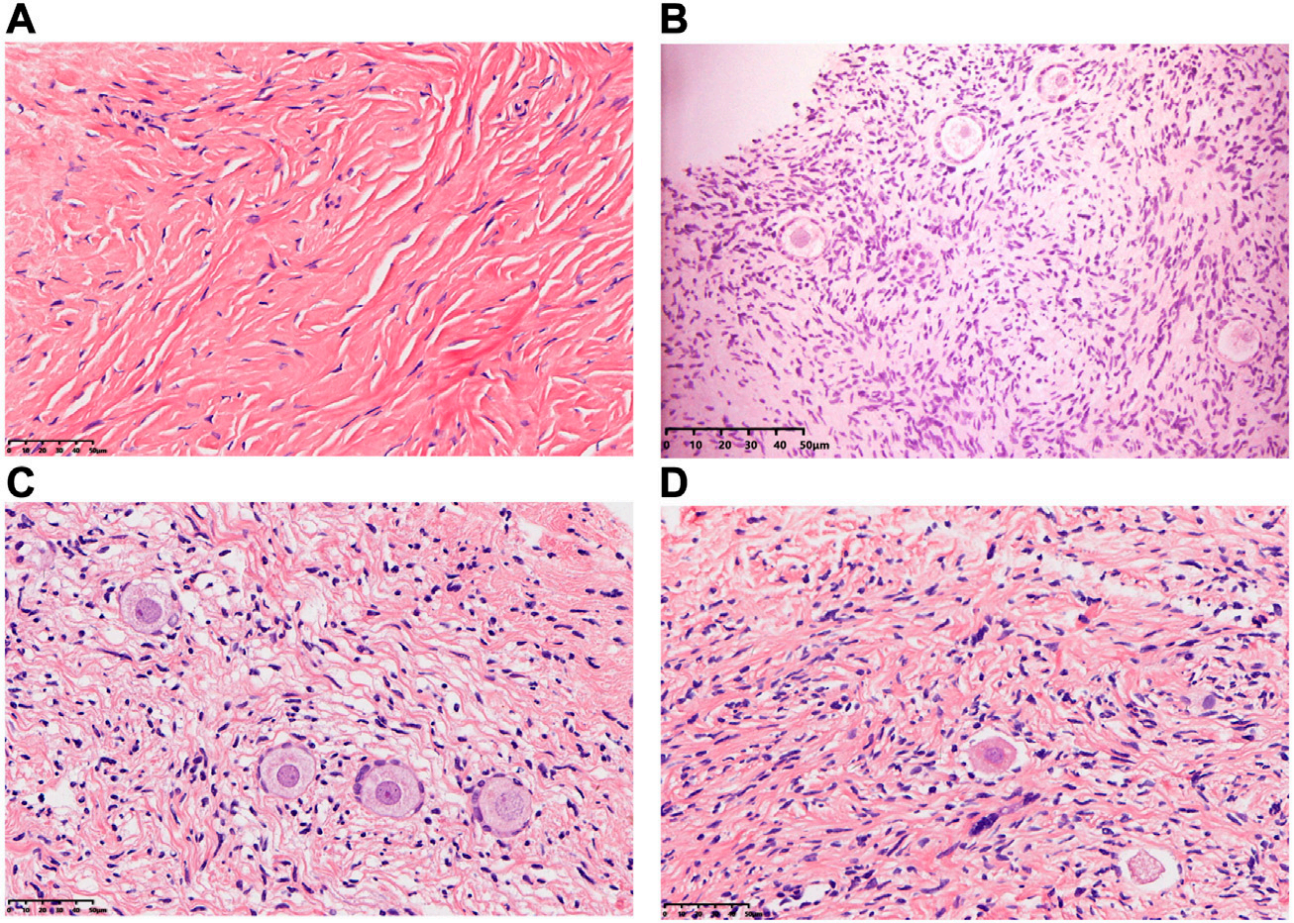
Morphology of human ovarian follicles in the CK and T groups. **(A,B)** Fresh human ovarian cortical tissue. **(C,D)** Human ovarian cortical tissue after vitrification/warming.

In comparison with the CK group, the morphology of most primordial follicles in the T-group was found to be in a well-preserved state (Figure 2), but vitrification can cause stromal cell edema in human ovarian cortical tissue. After HE staining, one HE staining sheet was selected from each patient to count the number of primordial and primary follicles under a ×20 objective microscope. The total numbers of follicles in the CK and T groups were 429 and 398, respectively. The normal rates of primitive follicles (CK group: 95%, T group: 80.4%) and primary follicles (CK group: 93%, T group: 78.7%) in the CK group were significantly higher than those in the T-group (Table 1).

**TABLE 1:** Impact of vitrification on the morphology of human ovarian follicles.

Group	Total number of follicles	Number of primordial follicles		Number of primary follicles	
		Total	Normal (%)	Total	Normal (%)
CK	429	364	345 (94.8%)	65	60 (92.3%)
T	398	351	282 (80.3%)	47	37 (78.7%)
P			**0.000**		**0.037**

Note: The bold values was *p* value and *p* value through Chi-square test as appropriate. *p* < 0.05 was considered statistically significant.

### 3.2 Assessment of apoptosis of the follicles in human ovarian tissue

**FIGURE 3:**
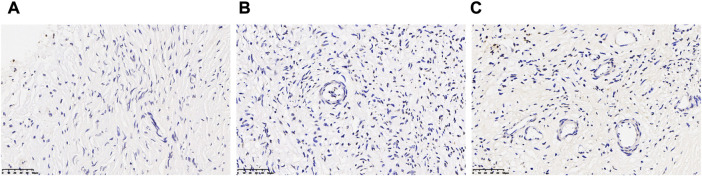
Assessment of apoptosis of the follicle in human ovarian tissue before and after vitrification. **(A)** Ovarian tissue in the CK group and a few apoptotic cells in the oocytes and granular cells. **(B,C)** Ovarian tissue in the T group and apoptotic cells were increased in the T group.

TUNEL analysis showed no apoptotic cells in oocytes and granular cells, and occasionally apoptotic cells in mesenchymal cells in fresh human ovarian cortical tissue. After vitrification/warming, very few apoptotic cells were found in the granular and mesenchymal cells in the T-group (Figure 3).

### 3.3 Data quality of RNA-seq

**FIGURE 4:**
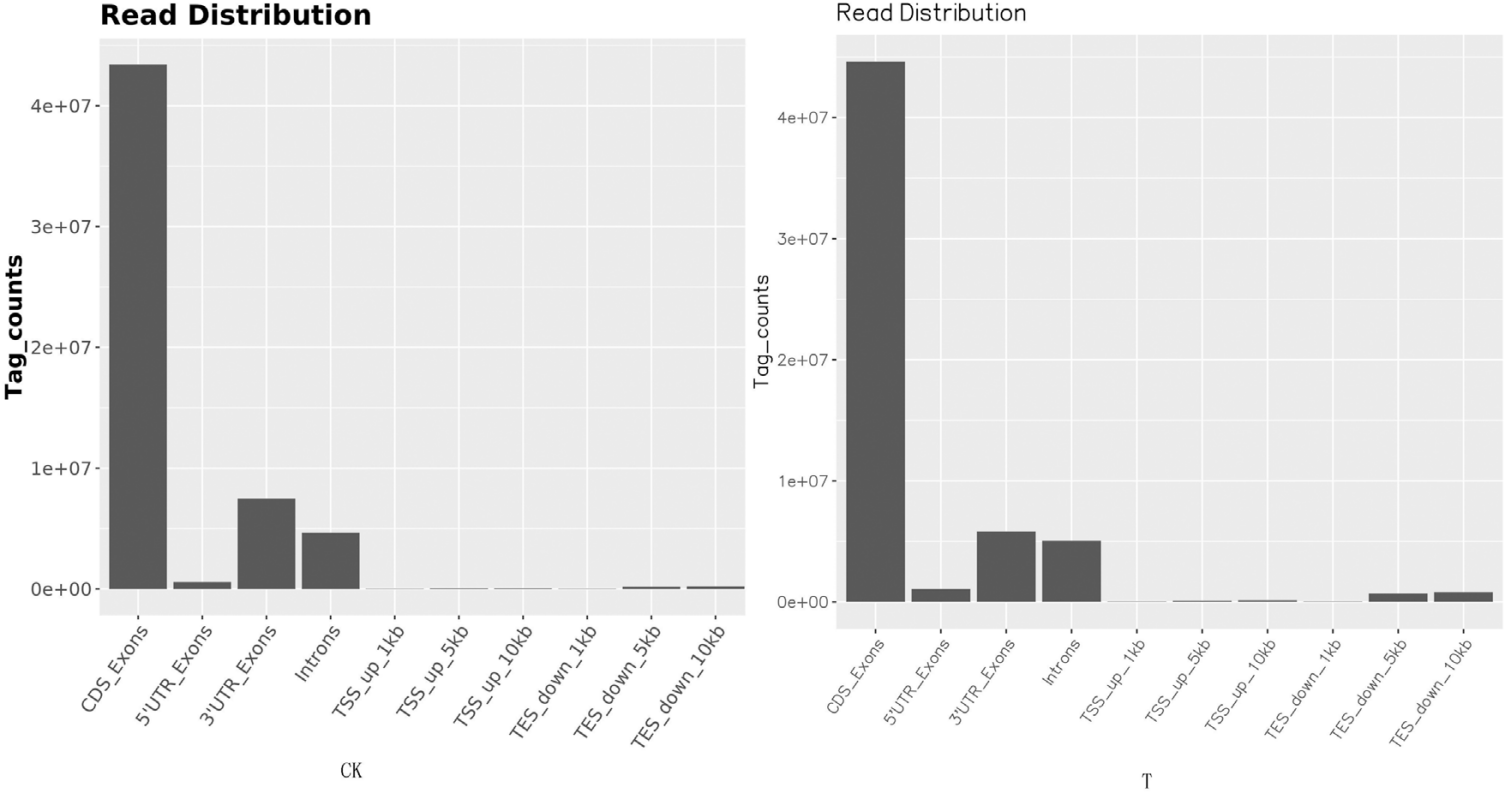
Read distribution map in the gene regions. The distribution of reads on the genome is statistically compared, and the localization regions are divided into CDS (the coding region), intron (the intron region), intergenic (the intergene region), and UTR (the 5′ and 3′ non-translating regions).

**FIGURE 5:**
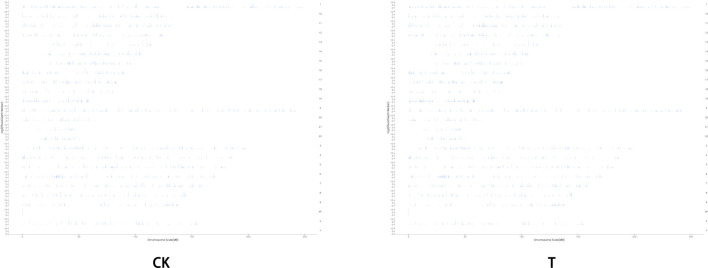
Pattern of read distributions on the chromosomes.

**FIGURE 6:**
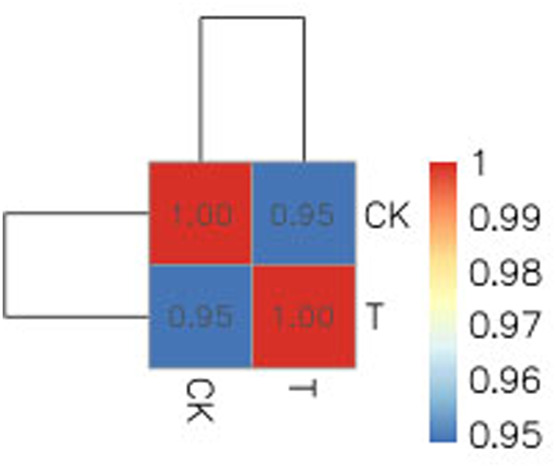
Correlation coefficients of the CK and T samples were 0.95 and 1.00, respectively.

Due to the small size of the human ovary cortex tissue, two RNA-seq libraries were established from 12 ovary tissue strips (six in the CK group and six in the T-group from four patients with endometrial cancer, one patient with cervical cancer, and one with vulvar cancer). More than 43 million raw reads were obtained from each RNA-seq library (Supplemental Table S2), which was sufficient for the quantitative analysis of gene expression. After filtering out sequencing adapters and low-quality reads, two RNA-seq libraries from the CK and T groups still generated over 40 million and 41 million clean reads, respectively (Supplemental Table S3). The mapped reads of each RNA-seq library were over 38 million, and multiple mapped reads and uniquely mapped reads were over 1.3 million and 36 million, respectively (Supplemental Table S4). The distribution of reads on the gene region and chromosome is shown in Figures 4, 5. The normalization of the expression level in each transcript was measured using FPKM, and genes with values of FPKM >1 were considered expressed. Therefore, 10,938 and 11,302 genes were detected as expressed in the CK and T groups, while the correlation coefficients of the two groups were 0.95 and 1.00, respectively (Figure 6). These results confirmed that the RNA-seq data met the conditions for differential expression analysis.

### 3.4 Differentially expressed genes of the human ovary tissues before and after vitrification/warming

**FIGURE 7:**
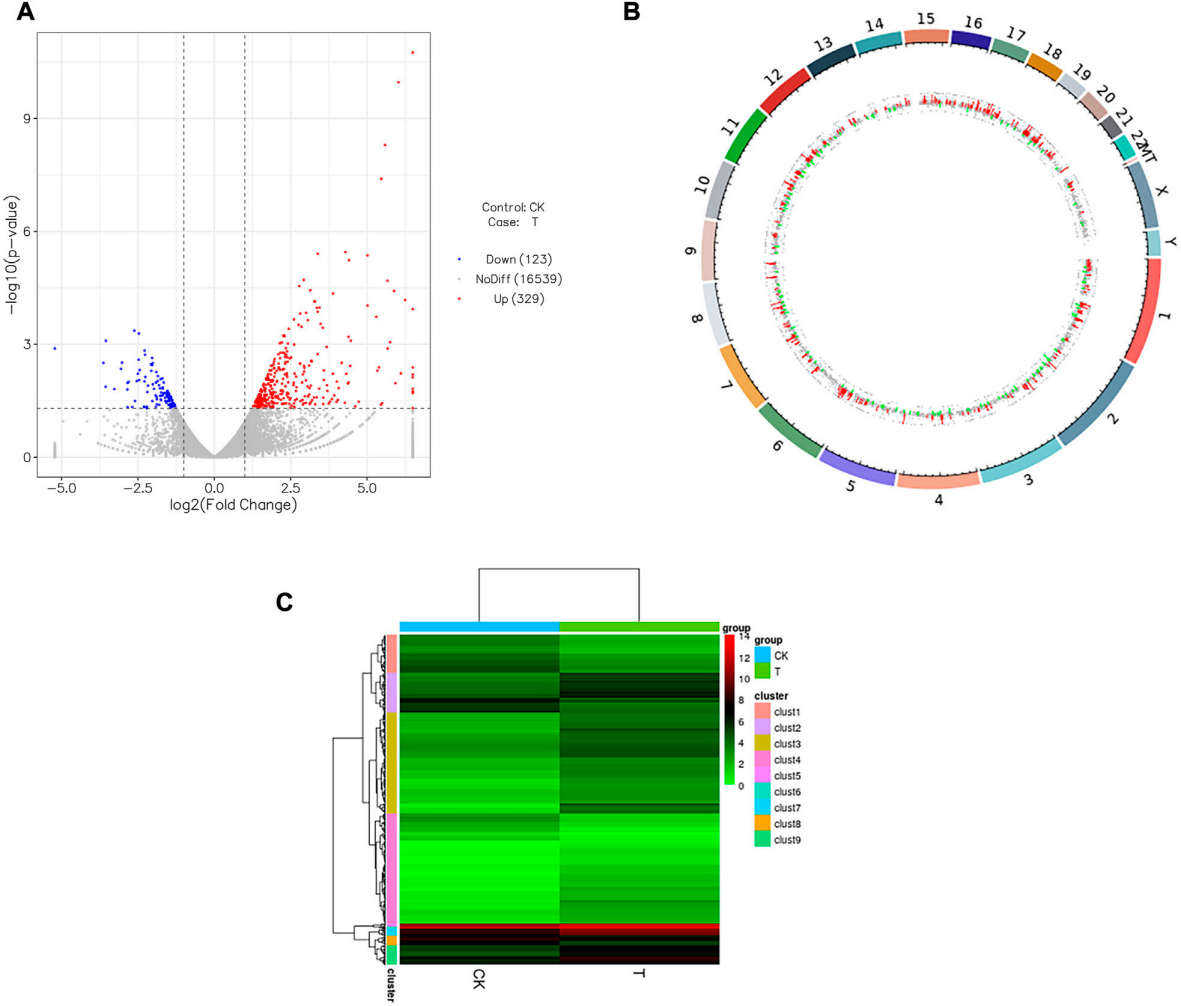
Influence of vitrification on the gene expression in human ovarian cortex. **(A)** Volcano plot showing differentially expressed genes (DEGs) meeting the conditions of |log2FoldChange| > 1 and *p* < 0.05. Red dots represent genes upregulated in vitrification (T) vs. fresh tissues (CK), while blue dots represent genes downregulated in T vs. CK, and gray dots represent the genes that were not differentially expressed in T vs. CK. **(B)** Outermost ring is the chromosomal band, which from the outside to the inside is the result of the differential expression analysis for differences. Red and green regions represent histograms of Iog2FoldChange values for up and downregulated genes, respectively, and the gray region represents a scatterplot of log2FoldChange values for gene expression without a difference in T vs. CK. **(C)** Heatmap of the clustering analysis (Euclidean method and complete linkage). Horizontal lines indicate genes, each column is a sample, red indicates high-expression genes, and green indicates low-expression genes.

As shown in Table 2, 452 significantly dysregulated genes (|log2FoldChange| > 1 and *p* < 0.05) were found between the CK and T groups. Among these, 329 were upregulated and 123 were downregulated genes. The details of each gene are available in supplemental material (Supplemental Table S6: upregulated genes; Supplemental Table S7: downregulated genes), and the differentially expressed gene (DEG) distributions for the two groups are shown as a volcano plot, heatmap, and genomeCircos images (Figures 7A–C)

**TABLE 2:** The total DEGs between CK and T groups.

Group		Up-regulated	Down-regulated	Total
CK	T	329	123	452

### 3.5 GO and KEGG pathway analysis of DEGs in human ovary cortex tissues before and after vitrification/warming

**FIGURE 8:**
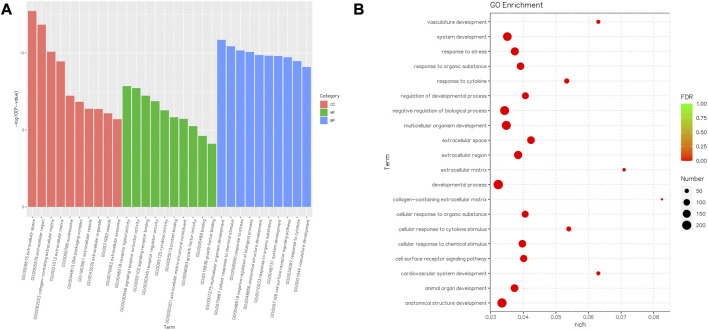
Barplot and Rich factor of GO enrichment. **(A)** Top 10 significantly enriched GO terms, including molecular function (MF) represented in green, cellular component (CC) represented in orange, and biological process (BP) represented in blue, are shown. The Y-axis represents the significance level as a *p*-value; the X-axis represents each name of the GO terms. **(B)** Rich factor refers to the ratio of the number of different genes enriched in the GO term compared to the number of genes annotated. The larger the Rich factor, the greater the degree of enrichment. FDR generally ranges from 0 to 1, and the closer to zero, the more significant the enrichment. The top 20 GO term entries are selected with the smallest FDR value, that is, the most significant enrichment.

The GO terms contain cellular component, molecular function, and biological process domains. The functional annotations of the DEGs of human ovary cortex tissues in the CK and T groups suggest that the GO terms were significantly enriched. A high proportion of genes were observed among the DEGs in the following categories: developmental process, anatomical structure development, negative regulation of biological processes, multicellular organism development, system development, response to stress, and the extracellular region (Figure 8).

**FIGURE 9:**
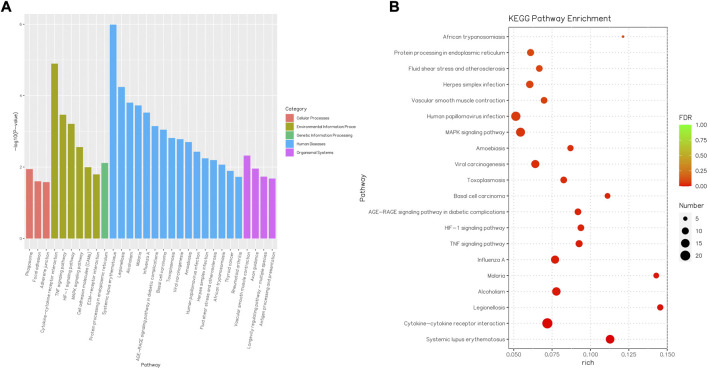
Barplot and Rich factor of KEGG enrichment. **(A)** Top 20 significantly enriched KEGG are shown. The Y-axis represents the significance level as a *p*-value; the X-axis represents each name of the pathway. **(B)** Rich factor refers to the ratio of the number of different genes enriched in the pathway to the number of genes annotated. The larger the Rich factor, the greater the degree of enrichment. FDR generally ranges from 0 to 1, and as it gets closer to zero, the more significant the enrichment. The top 20 KEGG pathway entries are selected with the smallest FDR value, that is, the most significant enrichment.

Consequently, the KEGG pathway analysis of DEGs for the CK and T groups is presented in Supplementary Tables S6, S7. A total of 372 genes were highly enriched for 43 pathways (*p* < 0.05), which were mainly related to systemic lupus erythematous, cytokine–cytokine receptor interaction, the TNF signaling pathway, the MAPK signaling pathway, and protein processing in the endoplasmic reticulum (Figure 9).

### 3.6 RT-PCR data validation

**FIGURE 10:**
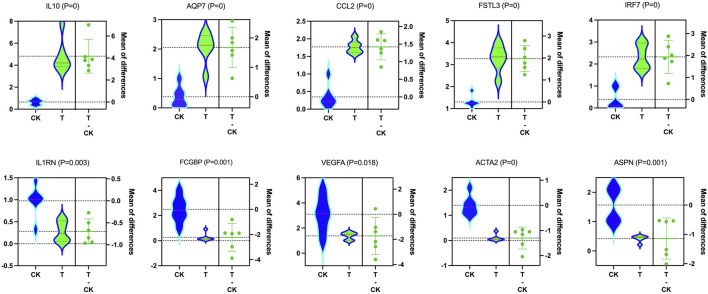
Molecular data validation. In validating the RNA-seq findings, qRT-PCR showed that IL10 (*p* = 0), AQP7 (*p* = 0), CCL2 (*p* = 0), FSTL3 (*p* = 0), and IRF7 (*p* = 0) were upregulated, while ILl RN (*p* = 0.003), FCGBP (*p* = 0.001), VEGFA (*p* = 0.018), ACTA2 (*p* = 0), and ASPN (*p* = 0.001) were downregulated considering T and CK.

The expression patterns of 10 dysregulated genes obtained using RT-PCR agreed with those observed in the RNA-seq analysis. *IL10*, *AQP7*, *CCL2*, *FSTL3*, and *IRF7* were significantly upregulated in vitrification/warming human ovary cortex tissues (*p* < 0.01), while *IL1RN*, *FCGBP*, *VEGFA*, *ACTA2*, and *ASPN* were significantly downregulated in the T-group (*p* < 0.05) compared to the CK group (Figure 10).

## 4 Discussion

OTC is a clinically approved technique in which the entire ovary or sections are harvested and frozen. After the completion of cancer therapy, these tissues are re-implanted (usually overlaid on the native ovary, fallopian tube, or peritoneum). This is the only option for prepubertal girls and can be performed immediately without hormonal stimulation. Re-implantation of ovarian cortex tissues can preserve not only fertility but also a normal hormonal milieu that may yield other health benefits such as improved bone and cardiovascular health and sexual function. This approach has resulted in pregnancy rates of approximately 30% and endocrine recovery in >90% of women [Fabbri et al., 2022; Arapaki et al., 2022; Dolmans et al., 2021].

Recently, several studies have used RNA-seq to measure the global transcriptomics of oocytes or follicles (Zhang et al., 2020; Jia et al., 2019; Huang et al., 2018; Wang et al., 2017; Liu et al., 2022). Pereira et al., 2020 reported that vitrification and auto-transplantation can affect the transcriptomic expression of mouse ovarian tissue. To confirm the safety of vitrified OTC, this study was the first (to the authors’ knowledge) to focus on mRNA transcriptomic changes that occur in human ovarian tissues after vitrification. Collective results have shown that vitrification can effectively preserve human ovarian tissue and effect the global transcriptome dynamics of human ovarian cortex tissues. Differences in transcriptomics, such as *TAC1, CHI3L1, HSPB1, AQP3, GPR3, ETV7, IL10, AQP7, CCL2, FSTL3, IRF7, IL1RN, FCGBP, VEGFA, ACTA2*, and *ASPN,* were observed.

Vitrification/warming is a simple, effective, and widely used protective measure for preserving oocytes, embryos, and ovarian tissues (Corral et al., 2018; Gu et al., 2020; Smith et al., 2010; Ting et al., 2012; Terraciano et al., 2020). It is well known that successful vitrification depends on a high concentration of cryoprotective agents (CPAs) and extremely rapid cooling and warming rates; however, CPAs can cause cytotoxicity in ovary tissues. Zhang et al. (2020) declared that a decreased concentration of CPAs could decrease the effect of vitrification on the mRNA transcriptome of bovine oocytes. Several synthetic polymers, including PVP and polyglycerol, have been added to vitrification solutions to improve the outcome of FP (Shahsavari et al., 2020). In this study, Cryo 3 in the Ova Cryo Kit Type M contained PVP. A total of 452 significantly dysregulated genes were observed in patients after OTC, and fewer than 623 gene expressions were significantly changed in vitrified mouse ovary tissues with ethylene glycol (EG) and dimethyl sulfoxide (DMSO) as CPAs.

Previous studies have shown that primordial follicles seem to be more resistant to cytotoxicity and cryodamage, whereas primary and secondary follicles are more susceptible to vitrification injuries. Some genes, such as *GDF-9, BMP-4/7/15, KITL, LIF*, and *FGF-2*, are related to primordial follicle growth (Celik et al., 2020). Liu et al. (2022) and Sang et al. (2021) demonstrated that genes, including *Lhx8, Nobox, Sohlh1, Tbpl2, Stk31*, and *Padi6*, might be biomarkers for predicting ovarian reserve. The present study showed that vitrification/warming procedures do not change the aforementioned genes and so these procedures may be a relatively effective method for protecting the ovarian reserve and egg quality.

In this study, most genes related to ovarian reserve and primordial follicle reactivation were not altered; however, a larger number of dysregulated genes were identified. Some studies have reported that *AQP1, AQP3*, and *AQP9* present in the ovarian follicle corpus luteum contribute to the rapid growth of follicles during folliculogenesis, follicular fluid accumulation, and nutrient supply to granulosa cells and oocytes (Lm et al., 2020). Sales et al. (2016) found that mRNA levels of *AQP3* and *AQP9* in bovine ovaries were downregulated, following vitrification, which can cause *AQP3* upregulation and *AQP1* mRNA downregulation in human ovary tissues, suggesting that *AQP1* and *AQP3* may have different functional mechanisms for human follicle growth.

Some studies have shown that human primordial follicles survive freezing and thawing quite efficiently with survival rates of more than 80%, whereas only 20–40% of the follicles survive after transplantation because of revascularization (Kristensen et al., 2018; Gavish et al., 2018), which is considered the biggest limitation to OTC. *ETV7, YPEL4*, and *CXCR4* have been identified as important transcription factors involved in hematopoiesis (Wu et al., 2020; Mattebo et al., 2021; Kawaguchi et al., 2019). *ETV7* and *YPEL4* were upregulated, and *CXCR4* mRNA was downregulated in human ovary tissues after vitrification, which may be a stress response to cryopreservation damage. Some dysregulated genes were related to embryo development (*H1, FOSL1, PHLDA2, SOX15,* and *RND1*), cell cycles (*CDC7*), cell proliferation (*LETM2*), and microtubules (*ARL4D* and *HSPB1*). Abdollahi et al. (2013) suggested that vitrification/warming affected the expression of some apoptosis-related genes (*Fas, caspase8*, and *BIRC5*). This study showed that vitrification can lead to changes in the expression of other apoptosis-related genes (*DUSP8* and *SPOCD1*) and that vitrification/warming has a minor effect on cell apoptosis in human ovarian tissue. These results also suggest that the detected dysregulated genes may not be critical for the progression of folliculogenesis and ovarian function, yet vitrification still affects the different cellular activities of various types of cells in the ovarian cortex tissue.

For vitrification, a two-step treatment would be effective in which ovary tissues are first pre-treated in a solution containing a low concentration of cryoprotectant for permeation under less toxic conditions and are then treated in a vitrification solution for a short time, which causes the cells to shrink by rapid dehydration. After warming, it is preferable to remove the permeated cryoprotectant from the cells. Regarding GO enrichment analysis, the extracellular space and regions were best when significantly enriched, which may have been due to shrinkage. Some genes were enriched in the extracellular space and region, like the GO terms such as *TAC1, CCL26, CXCL2, CCL2, SELE*, and *MSR1*. These results indicate that these genes may play specific roles in the extracellular space and regions. The cellular responses to chemical stimuli and responses to stress were significantly enriched, and this appearance indicates that the vitrification/warming procedure stimulated the ovarian tissue, and the ovarian response to these stimuli resulted in upregulation (*POLR2J, TAC1, PAX6*, and *BCL3*) or downregulation of genes (*CXCR4, SELE, STAB1*, and *SFRP1*).

Three pathways, including cytokine–cytokine receptor interaction, the MAPK signaling pathway, and the PI3K signaling pathway, were selected from the significantly enriched pathways and were associated with mammalian reproduction. Most of the DEGs participating in the cytokine–cytokine receptor interaction and the MAPK signaling pathway were upregulated, which indicated that these two pathways were activated in human ovarian tissues after vitrification/warming. MAPK and PI3K signaling pathways can regulate biological processes, such as fertilization, embryonic development, and oogenesis, while *SPOCD1* can activate the PI3K/AKT pathway to restrain cell apoptosis (Liu et al., 2020). DUSP8 is emerging as a critical negative regulator of the MAPK pathway and is involved in the cell oxidative stress response and cell apoptosis (Ding et al., 2019). Other signaling pathways were also identified in this analysis. These pathways, including the ECM–receptor interaction, focal adhesion, Jak-STAT, Wnt, and cell cycle are associated with reproduction. Therefore, the vitrification process can activate or deactivate these physiological functions. Once ovarian grafts were able to support complete oocyte growth, the dysregulation of genes could be associated with a compensatory mechanism to maintain ovarian viability after vitrification, which qualitatively did not impair oogenesis.

Young adult female patients expressed a good satisfaction rate with OTC, and their experiences were beneficial. However, the use of cryopreserved ovarian tissue remains low (Leflon et al., 2022), and so it is very important to study the safety of vitrification of human ovaries to improve its application. mRNA can be transformed into proteins, which are involved in various functions of tissues and cells. HE, TUNEL, electron microscopy, and hormone testing are all used to verify the effectiveness of vitrification in human ovarian tissue based on morphology and function but cannot accurately explain the safety of vitrification/warming technology. As a transcript product, the expression profile and amount of mRNA can reflect, to some extent, the expression of functional proteins, which explains the effectiveness and safety of vitrification technology in preserving ovarian cortical tissue at the molecular level. Our results confirm that vitrification can damage the expression of mRNA in human ovarian cortical tissue. However, mRNA with differential changes is not the most important gene affecting ovarian function and oogenesis and so it also indicates that human ovarian vitrification is a clinically feasible technology to preserve female fertility.

A limitation to this study was the small sample size. Another limitation was that only transcriptomic changes in human ovarian tissues were tested and different cell types were not considered. Finally, as important functional molecules, proteins are of great research value, especially those related to cell structure, cell function, and biological function. Therefore, it is also necessary to analyze the safety of vitrification in preserving human ovarian tissue using proteomics or other techniques. Furthermore, there are several levels at which cellular responses to stress or damage can be modulated. Alternatively, there is a balance among these dysregulated genes to promote ovarian function after vitrification, and future research should focus on gene or protein levels in response to vitrification protocols.

## 5 Conclusion

In conclusion, this study adopted a vitrification/warming method to preserve human ovarian cortex tissues. The results suggest that vitrification induces changes in the expression of a particular mRNA in human ovarian tissues, which acts as a mechanism for cellular structural maintenance or important biological processes. Thus, ovarian vitrification is a promising avenue for FP, and further molecular studies (including those involving protein levels) of human ovaries are needed to evaluate whether altered gene expression, due to vitrification/warming, could result in any downstream consequences.

## Data Availability

The raw sequence data reported in this paper have been deposited in the Genome Sequence Archive (Chen et al., 2021) in National Genomics Data Center (CNCB-NGDC Members and Partners, 2022), China National Center for Bioinformation/Beijing Institute of Genomics, Chinese Academy of Sciences (GSA-Human: HRA004080) that are publicly accessible at https://ngdc.cncb.ac.cn/gsa-human.
