# Supplementary material for: Impacts of vitrification on the transcriptome of human ovarian tissue in patients with gynecological cancer
Source: Front Genet. 2023 Mar 17;14:1114650. doi: 10.3389/fgene.2023.1114650 (PMC10063885; doi:10.3389/fgene.2023.1114650)
Supplement: Supplementary file 1 [file Table1.docx]

| Sample | Reads No. | Bases(bp) | Q30(bp) | N (%) | Q20 (%) | Q30 (%) |
| --- | --- | --- | --- | --- | --- | --- |
| CK | 43373318 | 6505997700 | 5977678549 | 0.000962 | 96.8 | 91.87 |
| T | 44143210 | 6621481500 | 6083815881 | 0.000941 | 96.76 | 91.87 |

**Supplemental Table S1.** The Raw Data of samples in CK and T groups.
