# Supplementary material for: Impacts of vitrification on the transcriptome of human ovarian tissue in patients with gynecological cancer
Source: Front Genet. 2023 Mar 17;14:1114650. doi: 10.3389/fgene.2023.1114650 (PMC10063885; doi:10.3389/fgene.2023.1114650)
Supplement: Supplementary file 3 [file Table2.docx]

**Supplemental Table S3.** The Clean Reads of samples in CK and T groups.

| Sample | Clean Reads No. | Clean Data (bp) | Clean Reads % | Clean Data % |
| --- | --- | --- | --- | --- |
| CK | 40503164 | 6075474600 | 93.38 | 93.38 |
| T | 41614052 | 6242107800 | 94.27 | 94.27 |
