# Supplementary material for: Impacts of vitrification on the transcriptome of human ovarian tissue in patients with gynecological cancer
Source: Front Genet. 2023 Mar 17;14:1114650. doi: 10.3389/fgene.2023.1114650 (PMC10063885; doi:10.3389/fgene.2023.1114650)
Supplement: Supplementary file 4 [file Table3.docx]

**Supplemental Table S3.** The RNASeq Map Statistics.

| Sample | Clean_Reads | Total_Mapped | Multiple _Mapped | Uniquely_Mapped |
| --- | --- | --- | --- | --- |
| CK | 40503164 | 38274389（94.50%） | 1349736（3.53%） | 36924653（96.47%） |
| T | 41614052 | 40020885（96.17%） | 2480424（6.20%） | 37540461（93.80%） |
